# Development of a refugee health assessment toolkit for specific populations to support primary care

**DOI:** 10.1093/eurpub/ckac131.243

**Published:** 2022-10-25

**Authors:** T Boshari, S Hassan, K Hussain, J Billett, S Garry, L Weil

**Affiliations:** 1 Public Health, London Borough of Newham, London, UK; 2 London Operations Team, Office for Health Improvement and Disparities, London, UK; 3 Public Health, London Borough of Southwark, London, UK; 4 Asylum Seekers and Refugees, Association of Directors of Public Health, London, UK

## Abstract

**Issue/problem:**

The United Kingdom (UK) hosts c.136,000 refugees and last year received the most asylum applications in two decades. Despite this, expertise in migrant health is not widespread in general practice, with few comprehensive toolkits available to support crucial initial health assessments of new arrivals.

**Description of the problem:**

A large influx of Afghan refugees entered the UK in autumn 2021. In London, primary care practitioners quickly identified a lack of readily accessible, comprehensive guidance to support them in conducting health assessments for arrivals with a complex range of needs. This was compounded by many in primary care having little or no experience of migrant health.

**Results:**

To address this gap in advice on conducting initial health assessments, a bespoke toolkit was created. The toolkit consolidated advice from a range of partners and resources: the UK Afghan migrant health guide, clinicians with humanitarian experience, front-line practitioners, Doctors of the World, and those leading on the health and public health response. The toolkit ensured greater consistency in the nature and content of assessments, considered not only primary needs but also broader wellbeing, and was responsive to both anticipated and known health priorities.

**Lessons:**

The initial health assessment toolkit for Afghan migrants was well received by frontline staff and has implications for international practice in other areas providing similar health support. The toolkit and associated supporting information has formed a template that can be rapidly adapted to suit emerging needs, as has been done for new arrivals from Ukraine. This work has fed into best practice by the UK National Asylum Steering Group and is to be a case study for a WHO project on country-specific health assessments.

**Key messages:**

• The toolkit is a proof of concept for partnership working towards holistic initial health assessments of new migrants in primary care, bringing together best evidence and pragmatic practice.

• This work has implications for other countries experiencing similar trends in migration and providing health support to an increasing number of new refugees.

